# ^18^F-FDG distribution pattern improves the diagnostic accuracy of single pulmonary solid nodule

**DOI:** 10.3389/fonc.2022.983833

**Published:** 2022-10-06

**Authors:** Nina Zhou, Annan Zhang, Hua Su, Wei Zhao, Nan Li, Zhi Yang

**Affiliations:** ^1^ Key Laboratory of Carcinogenesis and Translational Research (Ministry of Education/Beijing), Department of Nuclear Medicine, Peking University Cancer Hospital and Institute, Beijing, China; ^2^ NMPA Key Laboratory for Research and Evaluation of Radiopharmaceuticals (National Medical Products Administration), Department of Nuclear Medicine, Peking University Cancer Hospital and Institute, Beijing, China

**Keywords:** Heterogeneity, ^18^F-FDG, differential diagnosis, pulmonary nodules, masses

## Abstract

**Background:**

The main purpose is to explore the use of visual assessment of the heterogeneous distribution of ^18^F-FDG in single pulmonary solid lesions to differentiate the benign from the malignant.

**Methods:**

The 200 cases of pulmonary nodules or masses examined by ^18^F-FDG PET/CT were retrospectively analyzed. The heterogeneity of ^18^F-FDG distribution of the lesion was visually and quantitatively evaluated and the higher part of metabolism was observed and measured at the proximal or distal part to determine the lesion nature. The sensitivity, specificity, PPV, NPV, and accuracy of this method were calculated.

**Results:**

Total 171 pulmonary lesions showed heterogeneity of ^18^F-FDG uptake, including the 111 malignant and 60 benign. 54/60 (90.00%) benign lesions showed higher ^18^F-FDG uptake visually at distal part, while 104/111 (93.69%) malignant lesions showed higher ^18^F-FDG uptake visually at the proximal part. This visual method has good repeatability with a high kappa value (0.821, p<0.001). 52/60 (86.67%) benign lesions showed higher ^18^F-FDG uptake quantitatively at distal part, while 107/111 (96.40%) malignant lesions showed higher ^18^F-FDG uptake quantitatively at the proximal part. The sensitivity, specificity, PPV, NPV and accuracy of visual and quantitative methods were 93.69%; 96.40%, 90.0%; 86.67%, 94.55%; 93.04%, 88.52%; 92.86%, 92.40%; 92.98%, respectively (p<0.001). When combining the metabolic value and morphological characteristics of PET/CT with visual ^18^F-FDG heterogeneous features, the accuracy reached to 98.25%. The other 29 lesions (14.5%) with no heterogeneity were smaller (2.17 ± 1.06 vs 3.58 ± 1.48, P<0.001).

**Conclusions:**

Benign and malignant lung lesions showed different heterogeneity of ^18^F-FDG uptake. Lung cancer can be effectively distinguished from infectious or inflammatory lesions by this simple and convenient method.

## Background

The differential diagnosis of benign and malignant pulmonary lesions has always been the focus of clinical attention. Early identification of the nature is very important for clinical decision-making. For most patients with inflammation, the disease can be gradually absorbed by anti-inflammatory or anti-tuberculosis treatment; but for lung cancer patients, more aggressive surgical resection, radiotherapy and chemotherapy or targeted therapy are adopted, and the prognosis is poor. As part of the solitary pulmonary nodules (SPNs) lack the typical morphological characteristics, it is difficult to determine whether it is benign or malignant (1); thus, the differential diagnosis of SPNs has always been a clinical challenge. At present, most lung lesions are diagnosed by puncture biopsy, but due to the heterogeneity of time and space inside the tumor, puncture results are prone to be false positive or negative (2, 3). For some patients with pulmonary nodules, regular CT follow-up is used to dynamically monitor the changes of nodules. On the one hand, the radiation dose of the patients is increased, and on the other hand, patients with malignant lesions may miss the best treatment period (4). Research has shown that radiomics is valuable in the identification of benign and malignant lung nodules (5), but it is currently mostly concentrated in the research stage, and is not convenient for clinical application. Therefore, it is particularly urgent and important to select simple and effective examination methods to distinguish benign pulmonary lesions.

As a non-invasive systemic examination, the ^18^F-FDG PET/CT has high value in the staging, differential diagnosis and treatment evaluation of lung cancer (6). Previous studies have mostly focused on distinguishing lung cancer from other benign lesions by the level of metabolic value. Although the metabolic value of lung cancer is higher than that of benign lesions in general, there is a large overlap between the two (7), and the false negative rate and false positive rate are higher, which lead to unnecessary resection of benign lesions and malignant diseases will not be operated in time, thus delaying treatment (8). Although ^18^F-FDG PET/CT is still controversial in the differential diagnosis of benign and malignant lung lesions in general, it is undeniable that the uptake of ^18^F-FDG is indeed related to the biological aggressiveness and the degree of malignant proliferation. Therefore, the metabolic complexity of SPNs should be considered comprehensively (9). Some scholars have proposed the concept of relative activity distribution index (RAD) and found that benign and malignant SPNs have different physiological and metabolic characteristics. The preliminary conclusion of their study is that the cellular uptake of ^18^F-FDG activity in the proximal part of malignant SPN is higher than that in the distal part, and the uptake of ^18^F-FDG activity in the distal part of benign nodules is higher than that of the proximal part (10).This method adopts multiple quantitative indicators, and the clinical practice is complicated. In addition, there is a certain selection bias in this study, the lesions included are all large masses, so the applicability of this method has not been further discussed.

In this study, we selected pulmonary nodules and masses of different sizes with high metabolism, retrospectively analyzed the differences in the metabolic distribution of the two groups of benign and malignant lesions, and explored the applicability and differential diagnosis value of ^18^F-FDG metabolic distribution characteristics for high-metabolic lung masses.

## Materials and methods

This retrospective study was approved by the Medical Ethics Committee of our Hospital. Patients referred to ^18^F-FDG PET/CT examination for discrimination of benign and malignant lung nodule between October 2010 and December 2020 were selected. Inclusion criteria for the study participation include all of the following conditions:(a) Single solid lung nodule or mass. (b)^18^F-FDG uptake higher than mediastinal blood pool. (c) Pathology confirmed primary adenocarcinoma or squamous carcinoma, benign lesion confirmed by pathology or followed-up chest CT showed lesion shrinked more than 30% or disappeared. Patient with suspicious lymph node in mediastinal or hilar were not excluded. Additionally, patients with any of the following conditions were excluded: (a) Pure GGO/mixed GGO(b) Distant metastasis revealed by PET/CT. (c)^18^F-FDG uptake lower than blood pool. (d)Multiple hypermetabolic nodules or masses in both lungs. (e)A previous history of other cancer. (f) Obvious motion artifacts. 130 malignant lesions and 55 benign lesions were confirmed by the surgery or biopsy, and 15 benign lesions were confirmed by regular CT follow-up.

### Imaging

The patients were instructed to fast for 6 h before ^18^F-FDG injection. The ^18^F was manufactured by HM-20 medical cyclotron (Sumitomo Corporation, Japan), and its radiochemical purity was greater than 95% and the ^18^F-FDG was administrated intravenously according to the patient’s body weight (3.0-3.7 MBq/kg).In all cases, the serum glucose concentration met our institutional requirement (≤140 mg/dL).Imaging was performed using a PET/CT scanner (Biograph64, SIEMENS, Erlangen, Germany) operated in 3D Flow Motion (bed entry speed 1 mm/s) from the apex of the skull to the mid-thigh, with a PET axial field-of-view of 21.6 cm. The PET images were reconstructed by the TrueX + TOF method offered by the vendor. Low-dose CT scans were acquired in CARE Dose4D mode (120 kV, 3.0 mm slice thickness). Chest HRCT was taken to observe the details of the nodule: 120 KV, 250mAs, slice thickness of 1mm.The injected activity was 3.7 MBq/kg, and the time from injection to scan was about 60±10 minutes.

### Image analysis

Images were reviewed using our local picture archiving and communication system (PACS), by two accredited radiologists (8 and 10 years of clinical experience respectively). All PET/CT and chest HRCT scans were reviewed independently by the two authors who were blinded to the personal data of patients, and the following features on PET and HRCT were recorded: location (peripheral/central), diameter(long/short), SUVmax, suspected ipsilateral lymph node(yes/no). Diagnosis on ^18^F-FDG PET/CT were made by typical benign signs (central laminated or diffuse calcification, popcorn pattern of calcification, satellite nodules) or malignant signs (spiculated margin, air bronchogram, vessel convergence, pleural indentation, and lesion with thick wall cavitation).

### Assessment the heterogeneity of the ^18^F-FDG distribution inside the lesion

Heterogeneity refers to the inconsistency of ^18^F-FDG uptake within the nodule or tumor, which has been reported in the literature and may be related to the blood supply within the lesion (11). Localization along broncho vascular bundle on CT images, The PET grayscale was adjusted from high to low by visual interpretation to clearly evaluate if the uptake of ^18^F-FDG in the lesion was heterogeneous (yes/no). The nodule or mass was considered as a sphere or ellipsoid by the axial, sagittal and coronal CT. The area from the center of the nodule or mass to the ipsilateral hilar or bronchial vascular bundle is defined as the proximal end from transverse, coronal and sagittal positions, and the center to the side facing away from the hilar or bronchial vascular bundle is defined as the distal end. Observe whether there is heterogeneity in the uptake of ^18^F-FDG in the two halves of the lesion. If there is heterogeneity, finally evaluate whether the metabolic type of the lesion is proximal-dominated or distal-dominated. Lesions with no ^18^F-FDG uptake heterogeneous was defined as homogeneous. Diagnosis discrepancies between observers were resolved by consensus. The SUVmax of the distal and proximal parts of the mass was measured. The ratio of SUVmax at the proximal end of the mass to SUVmax at the distal end of the mass was calculated.

### Statistical analysis

Statistical Package for the Social Sciences (SPSS) version 20.0 was used for the analytical work. Data exhibiting normal distribution are expressed as the mean±± standard deviation, and comparison between variables uses independent sample T test. Categorical variables were analyzed through the chi-squared test. The sensitivity, specificity, PPV, NPV and accuracy were calculated. Diagnostic accuracy of PET/CT only, ^18^F-FDG visual distribution, PET/CT+^18^F-FDG visual distribution and ^18^F-FDG quantitative heterogeneous uptake were compared. The variability of the two observers was compared by Cohen’s kappa coefficient. The p <0.05 was considered statistically significant.

## Results

### The characteristics of patients

Total 200 patients with lung nodule or mass were included in the final analysis, including 130 cases of malignant tumor, 70 cases of benign lesion. Their median age was 60.82±10.67; (27-88) years. The malignant lesions were pathologically confirmed as adenocarcinoma (59, 45.4%), squamous cell carcinoma (63, 48.5%), small cell carcinoma (9, 6.9%). The benign lesions, confirmed by pathology or follow-up by chest CT, included tuberculosis (26, 37.1%), organizing pneumonia (38, 54.3%), granulomatous inflammation (4, 5.7%), inflammatory pseudotumor (2, 2.8%). The patient characteristics of this study were summarized in **Table 1**

**Table 1:** The characteristics of all patients.

Characteristics		Total (n = 200)	Malignant (n = 130)	Benign (n = 70)	P value
**Age (mean, range)**		60.82 ± 10.67 (27-88)	61.35 ± 10.22 (27-88)	59.84 ± 11.45 (30-85)	0.34
**Sex**	**Male**	134	88	46	0.78
	**Female**	66	42	24	
**Location**	**Centre**	53	43	10	0.004*
	**Periphery**	147	87	60	
**Size (short diameter)**		3.4 ± 1.5	3.8 ± 1.5	2.6 ± 1.1	< 0.001*
**SUVmax**		10.5 ± 5.9	12.9 ± 5.6	6.1 ± 3.4	< 0.001*
**Reference**					
**Pathology** **CT follow-up**		185 15	130 0	55 15	–

*P < 0.05.

### The results of heterogeneous distribution of ^18^F-FDG

#### Concordance between the two readers

The intra-observer variability in visual results by the two observers is shown in **Table 2**. The Cohen’s kappa coefficient demonstrated good agreement between the two observers’ visual evaluation (1: higher ^18^F-FDG uptake in proximal part, 2: higher ^18^F-FDG uptake in distal part, 3: homogeneous ^18^F-FDG uptake), with a kappa value of 0.821 (p<0.001). Evaluation discrepancies between observers were finally resolved by consensus. Total 171 lesions (111 malignant, 60 benign) had visual ^18^F-FDG uptake heterogeneity, and 29 lesions (19 malignant and 10 benign) showed no visual ^18^F-FDG uptake heterogeneity. The diameter in lesions showing ^18^F-FDG uptake homogeneity was shorter than that with heterogeneity (2.17 ± 1.06 vs 3.58 ± 1.48, P<0.001). The SUVmax in lesions with ^18^F-FDG uptake homogeneity was lower than that with heterogeneity (8.67 ± 5.25 vs 10.77 ± 6.07), but the difference was not statistically significant (P=0.08).

**Table 2:** The intra-observer variability in visual results by the two observers.

Reader 2	Reader 1			Total
	1	2	3	
**1**	101	0	0	101
**2**	4	58	0	62
**3**	14	3	20	37
**Total**	119	61	20	200

Kappa value=0.821(1: higher ^18^F-FDG uptake in proximal part, 2: higher ^18^F-FDG uptake in distal part, 3: homogeneous ^18^F-FDG uptake).

#### Heterogeneity of ^18^F-FDG distribution in malignant and benign lesions.

**Figure 1:**
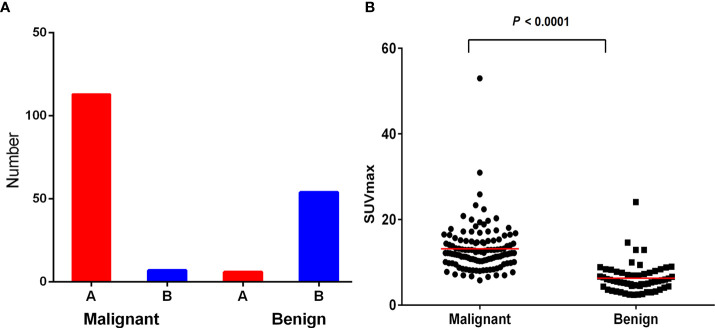
**(A)** Most malignant lesions showed higher ^18^F-FDG uptake in proximal part than that in the distal region. While most benign lesions showed higher ^18^F-FDG uptake in the distal region than that in the proximal region. **(B)** SUVmax of malignant lesion was higher than benign lesion (*P*< 0.000), but the SUVmax between the two groups overlaped greatly.

**Figure 2:**
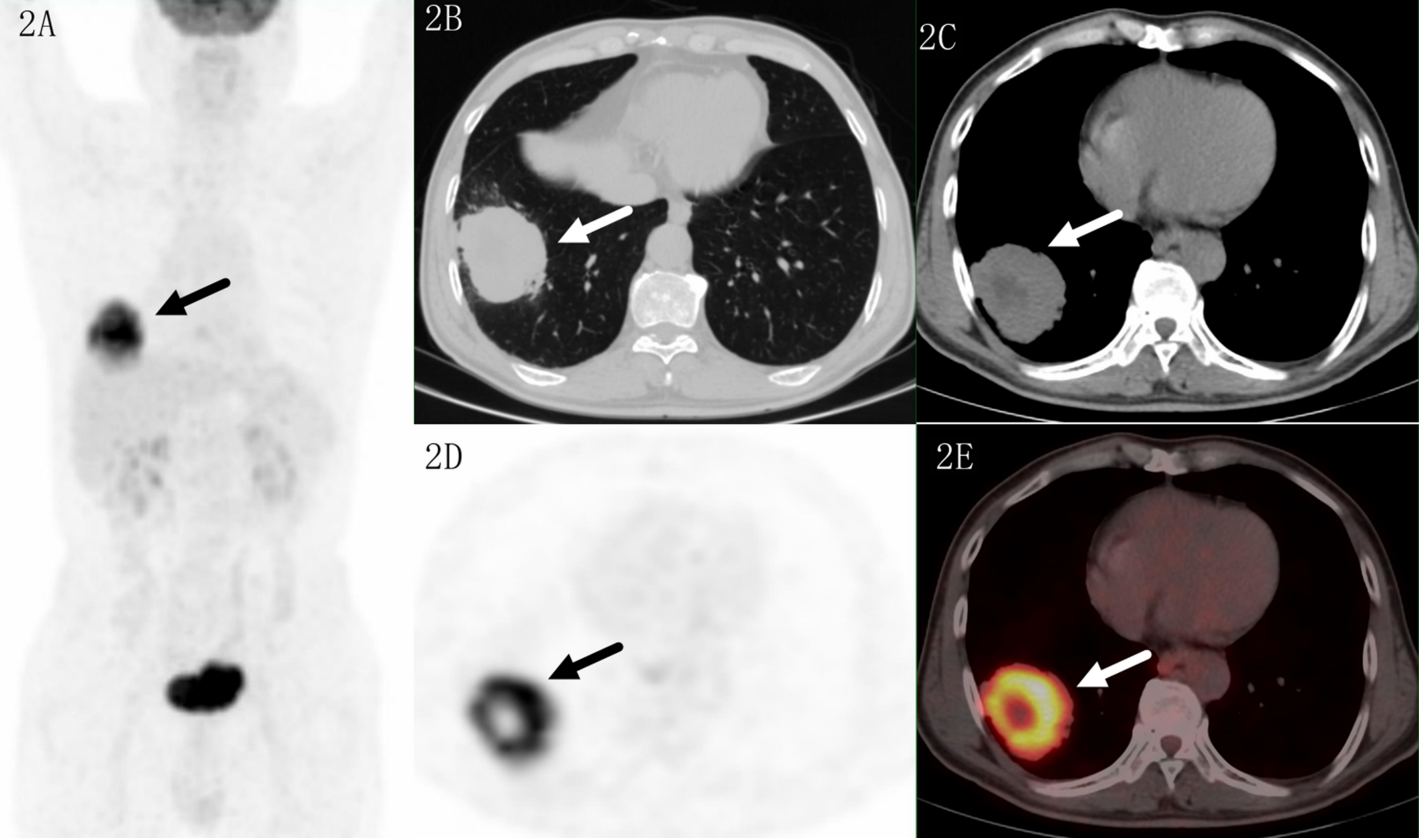
Images of a 69-year-old male patient with adenocarcinoma. PET MIP **(A)** showed a mass in the right lower lobe and a suspected lymph nodes in the right hilar. The lesion located in sub pleural site with a SUVmax of 16.4 on transaxial CT **(B, C)**, PET **(D)** and fused **(E)** images. Higher uptake was seen in the proximal region which indicated malignant lesion.

**Figure 3:**
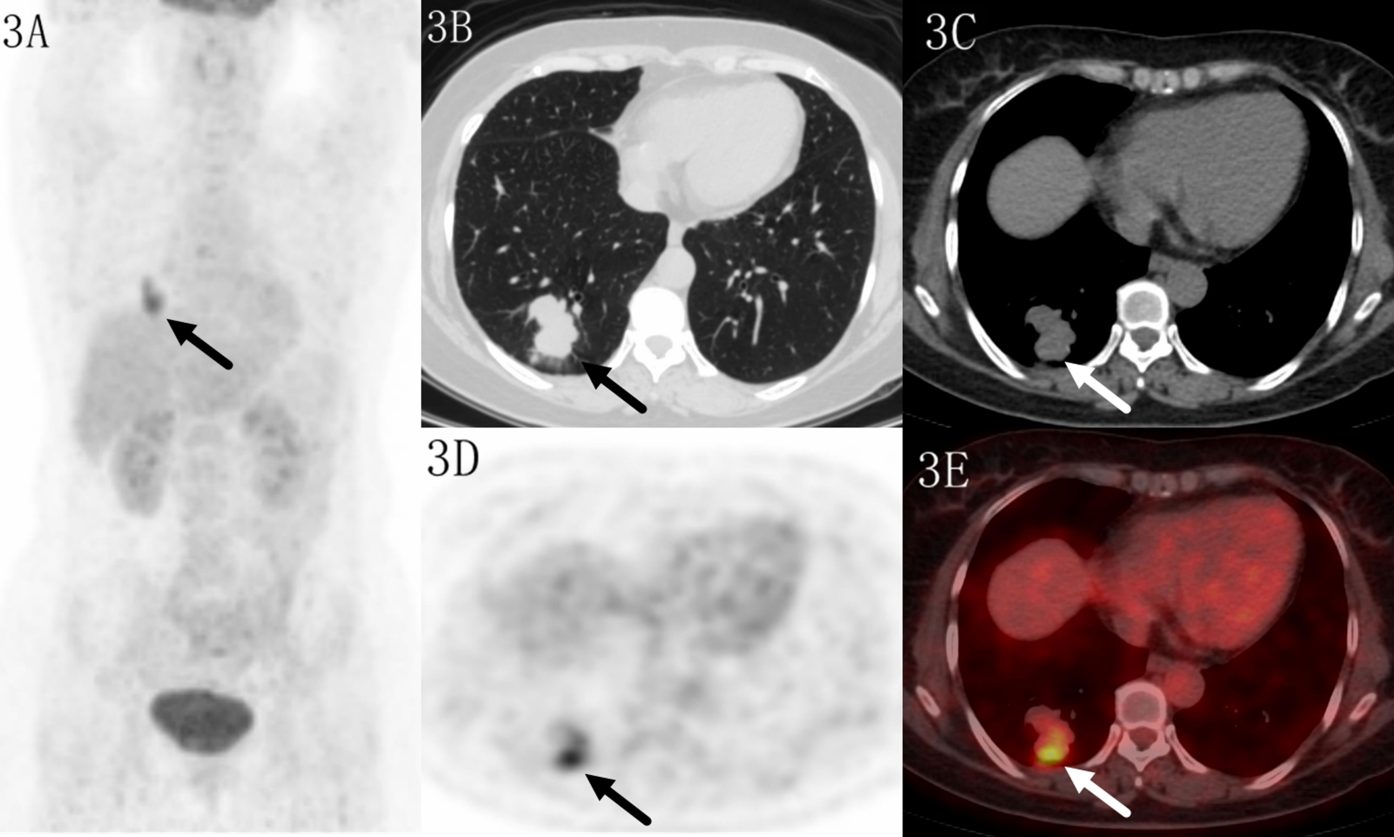
Images of a 52-year-old female patient with tuberculosis. PET MIP **(A)** showed a mass in the right lower lobe. The lobular lesion located in peripheral site, with a SUVmax of 6.6 on transaxial CT **(B, C)**, PET **(D)** and fused **(E)** images. Higher uptake was seen in the distal region which indicated benign lesion.

**Figure 4:**
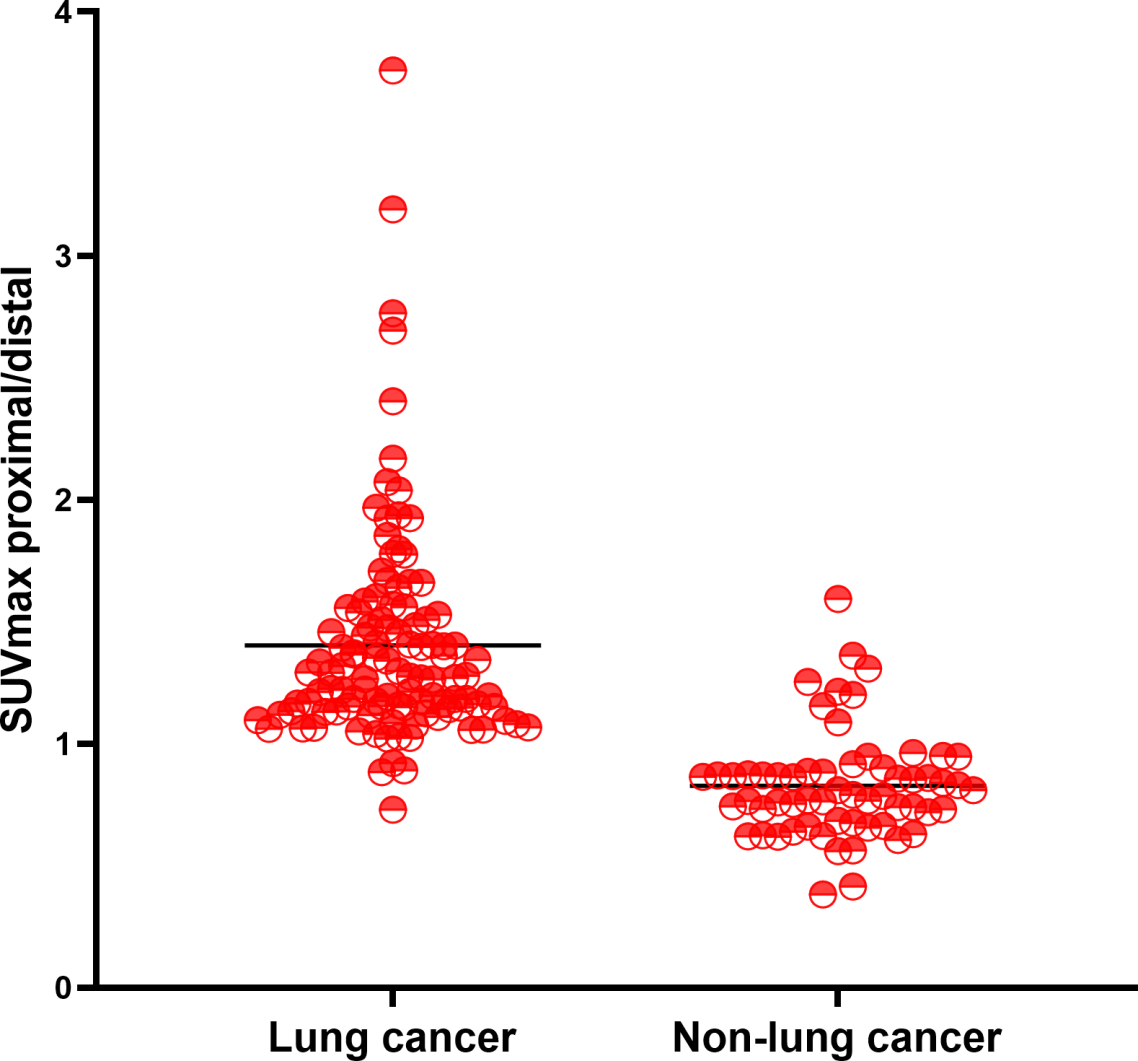
The ratio of SUVmax at the proximal end of the mass to SUVmax at the distal end of the mass.

Total 111 malignant and 60 benign lesions with ^18^F-FDG heterogeneity were used for further analysis. There were 104/111 (93.69%) malignant lesions with higher ^18^F-FDG uptake in proximal part than distal region. There were 54/60 (90.00%) benign lesions with higher ^18^F-FDG metabolic in the distal part than proximal part. (**Figure 1A**). SUVmax of malignant lesion was higher than the benign (13.19 ± 5.72 vs 6.29 ± 3.50, P<0.001), but the SUVmax between the two groups overlapped greatly (**Figure 1B**). Typical cases of malignant and benign were shown in **Figures 2**, **3**. There were 107/111 (96.40%) malignant lesions with higher SUVmax in proximal part than distal region. There were 52/60 (86.67%) benign lesions with higher SUVmax metabolic in the distal part than proximal part. The ratio of SUVmax at the proximal end of the mass to SUVmax at the distal end of the mass was shown in **Figure 4**.

### Diagnostic Accuracy of PET/CT only, visual distribution only, PET/CT+ visual distribution, and ^18^F-FDG quantitative heterogeneous uptake

The performance of ^18^F-FDG uptake visual and quantitative heterogeneity for distinguishing malignant from benign lesions were shown in **Table 3**. When higher ^18^F-FDG visual uptake in the proximal region was used for malignant diagnosis, the sensitivity, specificity, PPV, NPV, and diagnostic accuracy were 93.69%, 90.0%, 94.55%, 88.52%, 92.40%. Specificity, PPV and accuracy were higher than PET/CT (90.0% vs 50%, 94.55% vs 78.72%, 92.40% vs 82.46%). When combining PET/CT with ^18^F-FDG visually heterogeneous features, diagnostic accuracy reached to 98.25%. When higher SUVmax in the proximal region was used for malignant diagnosis, the sensitivity, specificity, PPV, NPV, and diagnostic accuracy were 96.40%, 86.67%, 93.04%, 92.86%, 92.98%. The area under the curve (AUC) of the ^18^F-FDG uptake visual heterogeneity, PET/CT, the combined index and ^18^F-FDG uptake quantitative heterogeneity for the differentiation of lung cancer from inflammatory disease were 0.92, 0.75, 0.97 and 0.93 (p<0.001, respectively), **Figure 4**.

**Table 3:** Diagnostic efficiency of different methods.

	TP	TN	FP	FN	Sensitivity	Specificity	PPV	NPV	Accurate	AUC	95%CI
PET/CT	111	30	30	0	100	50	78.72	100	82.46	0.75	0.87-0.97
FDG Heterogenity	104	54	6	7	93.69	90	94.55	88.52	92.40	0.92	0.66-0.84
Combining two methods	111	57	3	0	100	95	97.37	100	98.25	0.97	0.93-1.00
SUVmax proximal/distal	107	52	8	4	96.40	86.67	93.04	92.86	92.98	0.93	0.89-0.98

**Figure 5:**
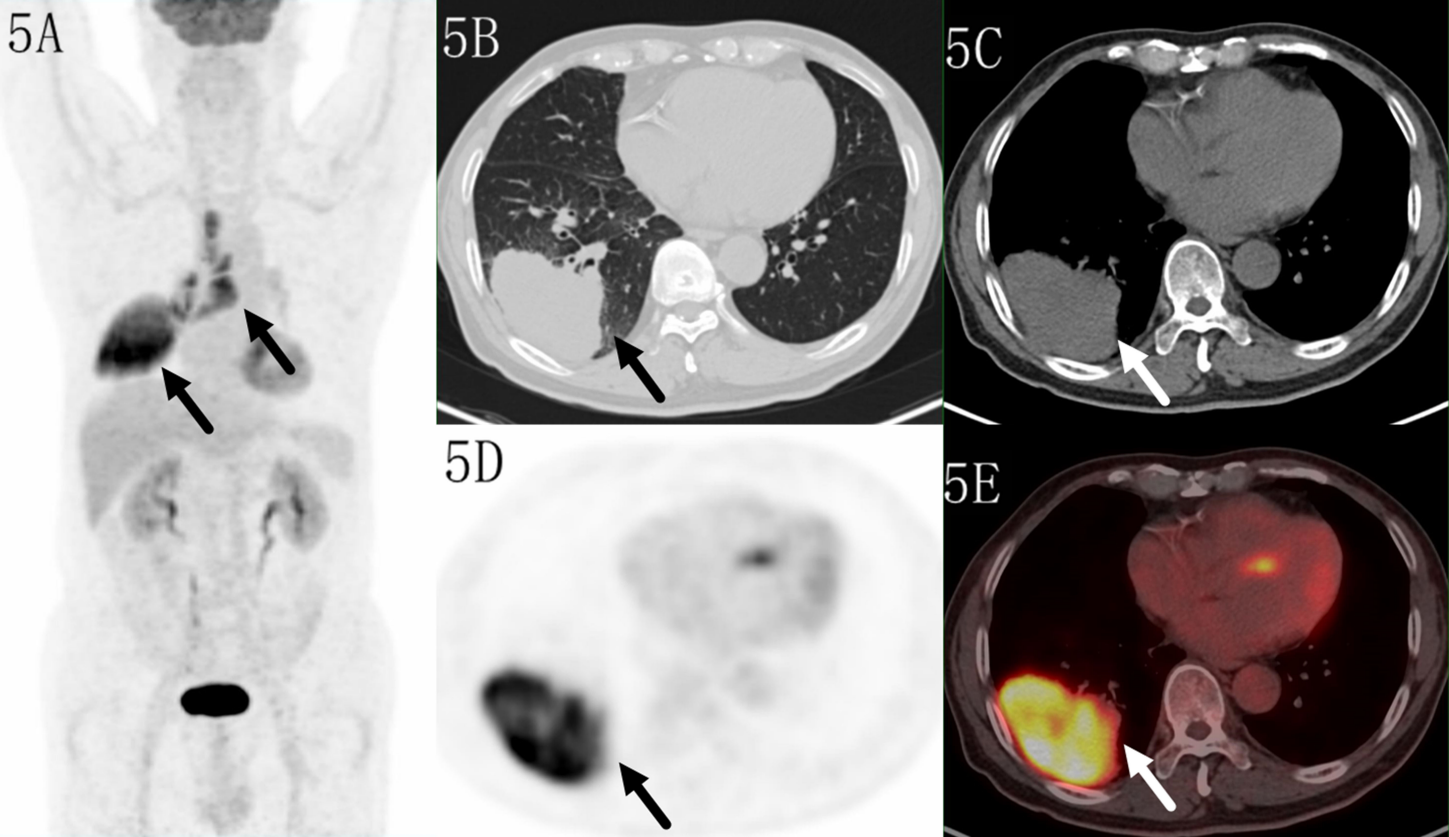
Images of a 70-year-old male patient with adenocarcinoma. PET MIP **(A)** showed a mass in the right lower lobe and multiple lymph nodes in the right hilar and mediastinum. The lesion located in subpleural site with a SUVmax of 14.4 on transaxialCT **(B, C)**, PET **(D)** and fused **(E)** images. Higher uptake was seen in the distal region.

**Figure 6:**
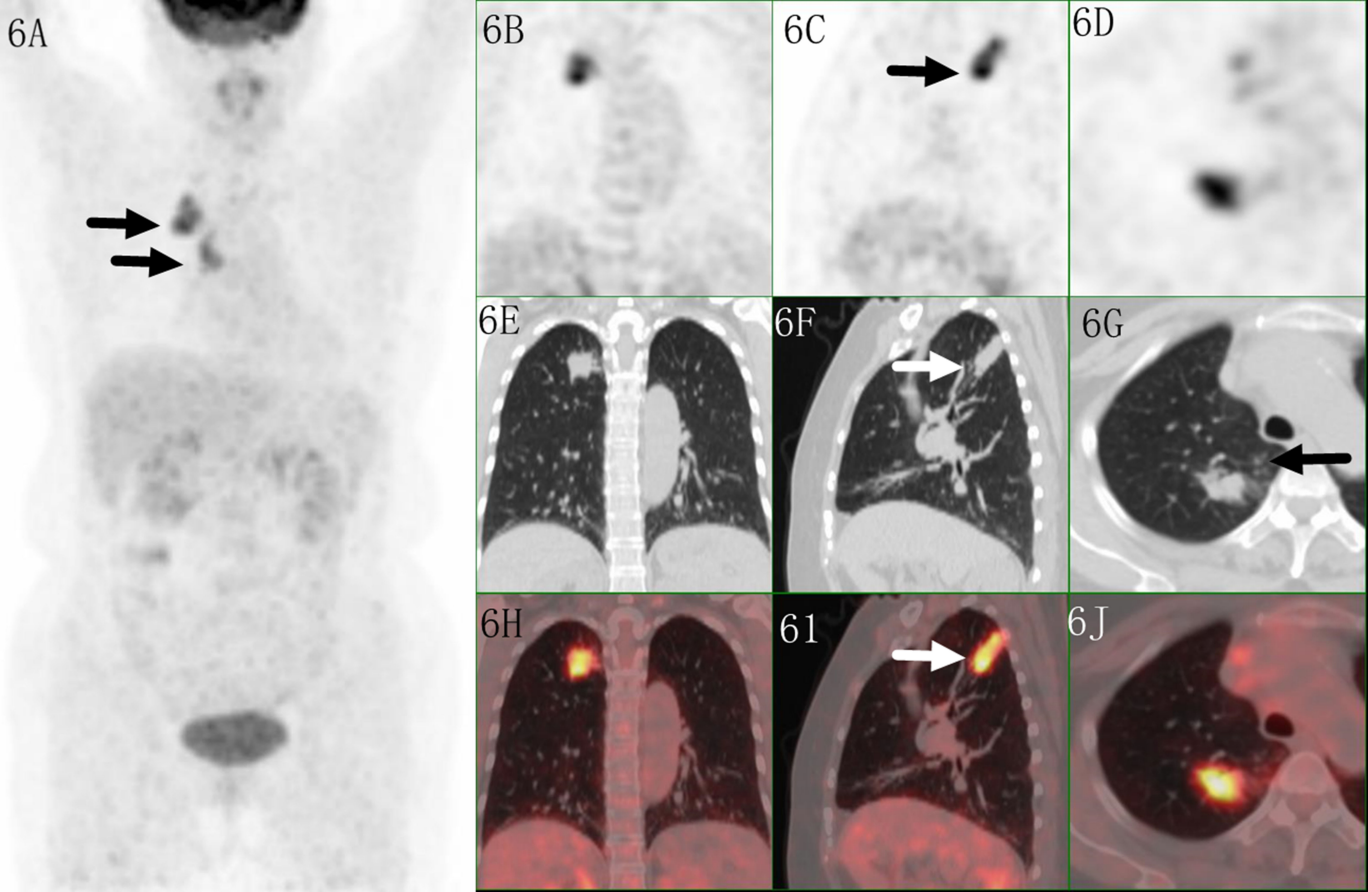
Images of a 61-year-old female patient with tuberculosis. PET MIP **(A)** showed a mass in the right upper lobe with mediastinal lymph node. The irregular lesion located in peripheral site, with a SUVmax of 8.9 on coronal/saggital/trans axial PET **(B–D)**, CT **(E–G)** and fused **(H–J)** images. Higher uptake was seen in the proximal region which indicated malignant lesion, but irregular shape with multiple small satellite nodules indicated tuberculosis.

**Figure 7:**
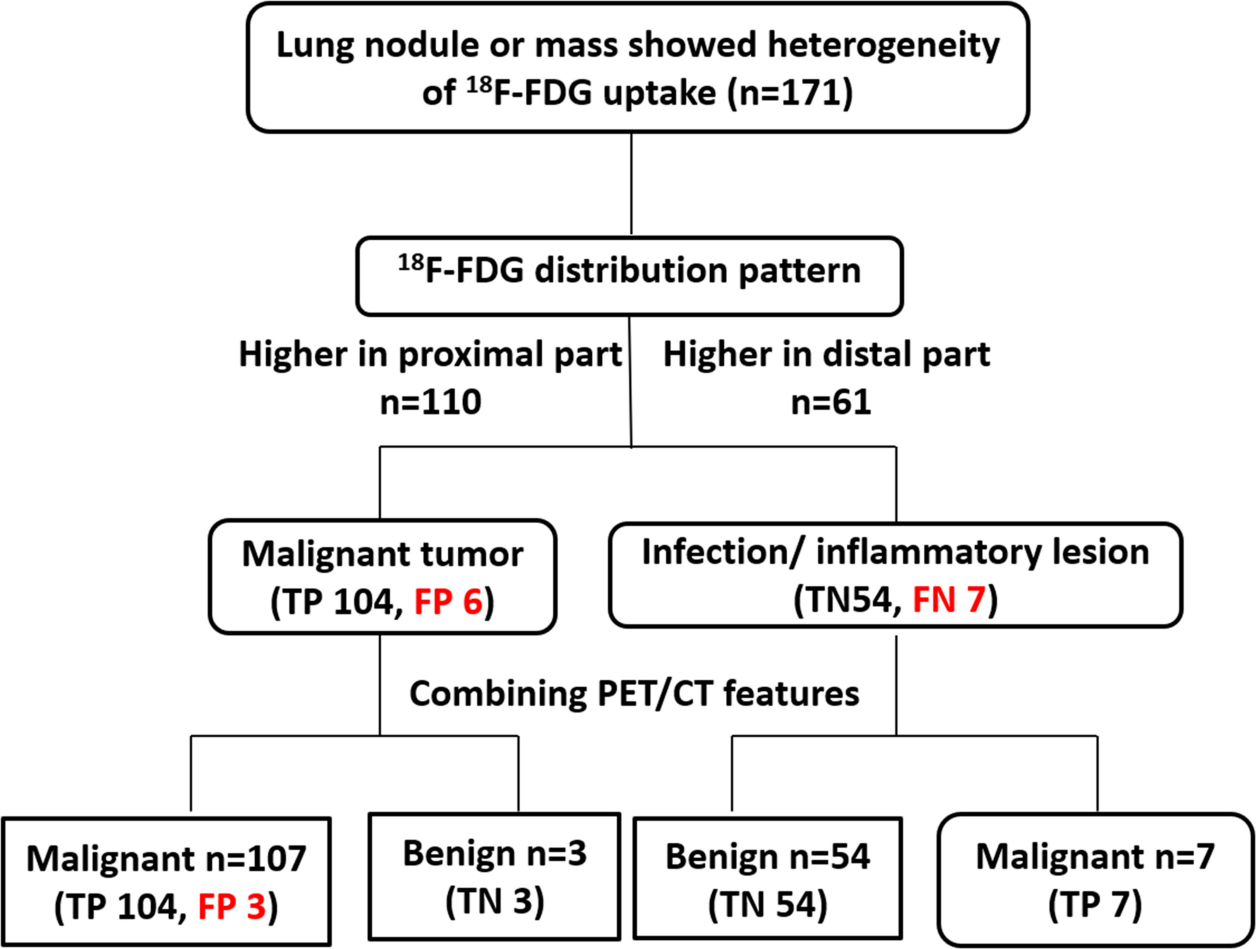
The proposed diagnostic chart flow. The lesions in 110 patients showed higher metabolism in the proximal end than in the distal end, and were initially judged to be malignant. Lesions in 61 patients showed higher distal metabolism than proximal and were thus judged to be benign. After all the lesions were further combined with PET/CT features, 3 of the malignant lesions were finally corrected to be benign, and the other 3 were still misdiagnosed as malignant. All 7 benign lesions were reclassified as malignant lesions. Based on the diagnostic chart flow, the diagnostic accuracy reached to 98.2%.

Only 7/111(6.3%) malignant lesions showed higher ^18^F-FDG uptake in distal part, including 3 adenocarcinoma and 4 squamous carcinomas, these lesions all located in subpleural sites (**Figure 5**), while combining CT feature, all lesions diagnosed as malignant tumor. Only 6/60 (10%) benign lesions showed higher ^18^F-FDG uptake in proximal part, including 4 tuberculosis, 1 granulomatous inflammation and 1 organizing pneumonia, while combining CT feature, it modified 3 lesions (**Figure 6**). These 7 false negative lesions and 6 false positive lesions were listed in **Table 4**. Based on the results, a diagnostic chart flow was proposed, it might enable us to distinguish benign lesion from malignant, following the chart flow, 168/171(98.25%) patients can be accurately diagnosed (**Figure 7**).

**Table 4:** Clinical characteristic and image features of 7 false negative lesions and 6 false positive lesions based on^18^F-FDG distribution pattern.

No.	Gender	Age	Short diameter	SUVmax	Higher ^18^F-FDG distribution	PET/CT Features	Diagnosis based on two methods	Pathology or follow up results
1	F	51	6.2	16.95	Distal	Malignant	Malignant	Squamous carcinoma
2	M	27	3.6	8.14	Distal	Malignant	Malignant	Adenocarcinoma
3	M	42	6.9	12.6	Distal	Malignant	Malignant	Squamous carcinoma
4	M	51	6.6	15.71	Distal	Malignant	Malignant	Squamous carcinoma
5	M	71	2.9	17.22	Distal	Malignant	Malignant	Squamous carcinoma
6	M	70	5.3	14.4	Distal	Malignant	Malignant	Adenocarcinoma
7	M	51	5.3	14	Distal	Malignant	Malignant	Adenocarcinoma
8	M	49	2.1	4.5	Proximal	Malignant	Malignant	Tuberculosis
9	M	55	3.6	12.9	Proximal	Malignant	Malignant	Tuberculosis
10	F	70	2.6	3.2	Proximal	Benign	Benign	Tuberculosis
11	F	48	1.6	2.5	Proximal	Benign	Benign	Granulomatous inflammation
12	M	56	3.4	7.04	Proximal	Malignant	Malignant	Organizing pneumonia
13	F	61	1.7	8.9	Proximal	Benign	Benign	Tuberculosis

## Discussion

This study found that ^18^F-FDG PET/CT has a certain value in the differential diagnosis of lung space-occupying lesions, especially benign and malignant SPN. The metabolic value of malignant lesions is higher than that of benign lesions, SUVmax (12.9 ± 5.6 vs 6.1 ± 3.4). Although it has a sensitivity of 100%, the specificity is relatively low, only 50%, which is in line with previous studies of 13% to 89% (12–16). It can be also seen from the metabolic imaging that there is a large overlap between the two groups. Studies have shown that inflammatory diseases of the lungs, including tuberculosis, cryptococcal pneumonia, and inflammatory pseudotumors are rich in inflammatory cells, such as leukocytes, macrophages and lymph node cells, similar with the tumor cells, and glucose transport receptors GLUT1–5 can be expressed on the surface, which promotes its high uptake of ^18^F-FDG (17, 18). All these indicate that it is not feasible to judge the benign and malignant pulmonary diseases based on the metabolic value alone. The high false positive rate makes some patients with benign lesions take unnecessary lobectomy or segmentectomy, which obviously affects the patient’s follow-up quality of life and causes unnecessary psychological burden. Therefore, it is important to find a simple and effective alternative way to distinguish the benign pulmonary lesions from the malignant.

The present study evaluated the strong practicality of heterogeneity of ^18^F-FDG distribution by visual and quantitative evaluation for distinguishing the malignant lung lesions from the benign. The advantage of visual method is that no special software is required, and it can be observed visually. Our study also confirmed that the accuracy of visual assessment method and quantitative measurement method are basically consistent, with accuracy of 92.40% and 92.98%. It is simple and easy to perform in the diagnosis process, and is especially suitable for high metabolic case that is prone to false positives. Of the 200 lung lesions in this study, 171 showed heterogeneous ^18^F-FDG uptake, with an effective rate of 85.5%. The result indicated that ^18^F-FDG uptake in the proximal area of lung cancers was significantly higher than the distal part; in contrast, the uptake of ^18^F-FDG in benign lesions in the distal was higher than the proximal. Furthermore, this method demonstrated better diagnostic efficacy than ^18^F-FDG PET/CT, with higher. The specificity, PPV and accuracy were higher than PET/CT (90.0% vs 50%, 94.55% vs 78.72%, 92.40% vs 82.46%). Combining the above two methods, the diagnostic accuracy can be further improved to 98.25%, which was not researched by previous study.

This method also has good reproducibility, and shows high evaluation consistency between two observers, with a high Kappa value of 0.821, which should be considered as a valuable investigative addition in the assessment of lung lesions. A similar study has proposed to use the relative activity distribution (RAD) index to identify benign and malignant SPNs. The RAD of malignant lesions (0.98 ± 0.03) was lower than that of benign lesions (1.01 ± 0.02), with RAD<1 as the standard, the specificity, accuracy, PPV, NPV reached 96.67%, 93.71%, 98.15%, 86.57% (10). Lin et al. found that pulmonary benign and malignant lesions have distinct ^18^F-FDG metabolic spatial distribution characteristics (FMSD). The FMSD in the proximal region of malignant lesions was significantly higher than that of the distal region, and the FMSD in the proximal region of benign lesions was significantly lower than that of the distal region. The sensitivity, specificity, PPV, NPV, and diagnostic accuracy of FMSD were 92.8%, 72.9%, 85.7%, 86.1%, and 89.2% (19), which is very similar to our research and further supports our conclusion, but diagnostic accuracy of combining PET/CT features with FMSD, and the optimal size of lesion for FMSD application was not discussed in their study. In our study, there were 29 patients whose lesions did not show the heterogeneity of ^18^F-FDG uptake. It was found that although the metabolism of these lesions was lower than that of patients with heterogeneous ^18^F-FDG uptake, they did not show statistical differences. The diameter of lesions in the 29 patients (10 cases benign, 19 cases malignant) is significantly smaller than the lesions of the rest 171 patients (p<0.001). The author speculates that the lesions of these patients may not show visual differences in metabolism due to their small diameter which indicated that this method were more suit for lesions larger than 2 cm.

Blood supply is considered to be one of the important reasons for the difference of metabolic distribution between benign and malignant lesions. Tumor is vascular dependent disease, and angiogenesis causes changes in blood volume, perfusion volume and capillary permeability (20). Active inflammatory nodules can cause changes in lumen size and capillary permeability, which determines the pathological basis of different blood flow patterns between tumor and inflammatory nodules. The blood supply of lung cancer is mainly by bronchial artery originating from descending aorta. In the malignant lesions, blood supply from the bronchial artery was more abundant, causing the tumor cells in the proximal growing and metabolizing actively, and ^18^F-FDG uptake was increased (21). However, chronic inflammation is more likely to stimulate the blood supply of extrapulmonary systemic circulation arteries, which are mostly located at the distal end of the heart (19). Therefore, the exudation of inflammatory lesions is more frequent at the distal end, leading to the high ^18^F-FDG uptake in this location. Among the 7 lung cancers with the distal end higher ^18^F-FDG uptake than the proximal, all lesions located in sub pleural and 5 cases with pleural invasion. Based on this, it is speculated that the blood supply of this part of the disease may be mainly related to the extracorporeal pulmonary circulation, because this part of the blood vessel (intercostal artery) is mainly involved in the blood supply of the pleura and chest. Similarly, among the 6 benign lesions in which the ^18^F-FDG uptake was higher at the proximal end than the distal, 4 of the lesions were central, which may be related to the involvement of the bronchial arteries near the hilum for blood supply. However, the metabolism of lesions may be affected by multiple factors (11), and the vascular apparatus may be one of the factors, while the other factors need to be further studied by subsequent large samples, multi-centers and combined with pathology.

The innovation of this study is to propose a new indicator of ^18^F-FDG PET/CT: the characteristics of the metabolic distribution of the lesions, which have high accuracy in identifying high metabolic occupying lesions, can reduce the false positives of PET/CT diagnosis, and it has good reproducibility, suitable for larger and unevenly metabolized lesions. However, for some small or visually more uniformly metabolic cases of lesion, the applicability of this method is poor and further large-sample prospective studies are needed in the future to verify. So far, the mechanism that produces the different metabolic distributions of benign and malignant lesions has not been supported by pathological mechanism. It needs to be further confirmed by large-scale prospective studies, animal model experiments, and pathological (for example, blood vessel density) control studies. Another limitation of this paper is that visual evaluation is a subjective evaluation index, which may be related to the experience of the doctor.

## Conclusions

In summary, the benign and malignant lung lesions show different ^18^F-FDG metabolic distribution patterns. The ^18^F-FDG uptake of lung cancer at the proximal end is higher than the distal, and the metabolic distribution of inflammatory lesions is mostly the opposite. The metabolic distribution characteristics of ^18^F-FDG can provide a reference for the identification of high lung metabolism and reduce false positives. Because of its simplicity and convenience, it is worthy of further promotion and application in clinical practice.

## Data availability statement

The original contributions presented in the study are included in the article/supplementary materials, further inquiries can be directed to the corresponding author/s.

## Ethics statement

The studies involving human participants were reviewed and approved by the Peking University Cancer Hospital and affiliation of ethics committee. The patients/participants provided their written informed consent to participate in this study.

## Author contributions

All authors listed have made a substantial, direct, and intellectual contribution to the work and approved it for publication.

## Funding

The current research was financially supported by the Science Foundation of Peking University Cancer Hospital (No.2021-4) and Beijing Hospitals Authority Dengfeng Project (No. DFL20191102)

## Conflict of interest

The authors declare that the research was conducted in the absence of any commercial or financial relationships that could be construed as a potential conflict of interest.

## Publisher’s note

All claims expressed in this article are solely those of the authors and do not necessarily represent those of their affiliated organizations, or those of the publisher, the editors and the reviewers. Any product that may be evaluated in this article, or claim that may be made by its manufacturer, is not guaranteed or endorsed by the publisher.

## Abbreviations

**PET,** Positron Emission Tomography; **CT,** Computed Tomography; **SUVmax,** the maximum single-voxel standardized uptake value; **ROIs,** Regions of interest; **FOV,** Field-of-view; **NPV,** negative predictive value, **PPV,** positive predictive value, **TP,** true positive, **FP,** false positive, **TN,** true negative, **FN,** false negative.
